# Recent Trends in Computational Biomedical Research

**DOI:** 10.3390/life12010027

**Published:** 2021-12-24

**Authors:** Md. Altaf-Ul-Amin, Shigehiko Kanaya, Naoaki Ono, Ming Huang

**Affiliations:** Division of Information Science, Nara Institute of Science and Technology, Ikoma 630-0192, Japan; skanaya@gtc.naist.jp (S.K.); nono@is.naist.jp (N.O.); alex-mhuang@is.naist.jp (M.H.)

Recent advances in information technology have brought forth a paradigm shift in science, especially in the biology and medical fields. Statistical methodologies based on high-performance computing and big data analysis are now indispensable for qualitative and quantitative understanding of experimental results. In fact, the last few decades have witnessed drastic improvements in high-throughput experiments in health science, for example, mass spectrometry, DNA microarray, and next-generation sequencing. Those methods have been providing massive data involving four major branches of omics (genomics, transcriptomics, proteomics, and metabolomics). On the other hand, cell imaging, clinical imaging, and personal healthcare devices are also providing important data concerning the human body and disease. In parallel, various methods of mathematical modeling such as machine learning have also developed rapidly. All of the types of these data can be utilized in computational approaches for biomedical research such as on understanding disease mechanisms, diagnosis, prognosis, drug discovery, drug repositioning, disease biomarkers, driver mutations, copy number variations, disease pathways, and much more. Therefore, the range of topics under “Recent Trends in Computational Biomedical Research” is extensive, and the present Special Issue is not a comprehensive representation of the subject. Nonetheless, the articles selected for this Special Issue represent a variety of topics relating to the title, and we are sharing with the readers with pleasure.

In this Special Issue, we published a total of eight good papers. Overall, four papers are in cardiovascular-related topics, two are linked to drug development, and the other two are on finding associations between genome sequence aberrations and diseases.

The paper titled “SMCKAT, a Sequential Multi-Dimensional CNV Kernel-Based Association Test” is on copy number variants (CNVs) [1]. Associations between CNVs and various diseases have been well studied before, and the current paper proposes a method called SMCKAT to test the association between the sequential order of CNVs and diseases. SMCKAT was evaluated by applying on CNV data sets, demonstrating its ability to exhibit rare or common CNVs by detecting specific biologically relevant chromosomal regions supported by the biomedical literature.

The paper with the title “The Genome-Wide Scanning of Potential Hotspots for Adenosine Methylation: A Potential Path to Neuronal Development” is about the methylation of adenosines at the N6 position (m6A) [2]. This methylation is the most frequent type of internal modification in mRNAs and is attributable to diverse roles in physiological developments and pathophysiological processes. This work applied a sliding window technique to identify the consensus site and annotated all m6A hotspots in the human genome. Most of the hotspots were found to be located in the non-coding regions, suggesting that methylation occurs before splicing. The role of this methylation in neuron physiology was also elaborated

An automatic ECG quality assessment method has been presented in the paper titled “Electrocardiogram Quality Assessment with a Generalized Deep Learning Model Assisted by Conditional Generative Adversarial Networks” [3]. This automatic method can help cardiologists perform diagnosis much faster. The proposed system first trained a conditional generative adversarial network model for data augmentation and then developed a deep quality assessment model based on a training dataset composed of real and generated ECG. The proposed system demonstrated improved performance in the ECG quality assessment and has the potential to be utilized in clinical practice.

The paper titled “A Maximum Flow-Based Approach to Prioritize Drugs for Drug Repurposing of Chronic Diseases” is on drug repurposing, i.e., using existing drugs to treat new/other diseases [4]. The work proposes a maximum-flow-based approach using protein–protein interactions (PPIs) of drug targets (proteins) and risk genes corresponding to chronic diseases such as breast cancer, inflammatory bowel disease (IBD), and chronic obstructive pulmonary disease (COPD). The top candidate drugs identified by the maximum flow-based approach were found to be experimentally validated by other independent studies.

Models and results of in silico prediction of the interactions between compounds of Jamu herbs and human proteins have been presented in the paper titled “Identification of Targeted Proteins by Jamu Formulas for Different Efficacies Using Machine Learning Approach” [5]. Verifying the proteins that are targeted by natural compounds is helpful to select natural herb-based drug candidates and to explain the scientific mechanisms of traditional medicines. 

The paper titled “Shared Molecular Mechanisms of Hypertrophic Cardiomyopathy and Its Clinical Presentations: Automated Molecular Mechanisms Extraction Approach” is about finding molecular mechanisms of hypertrophic cardiomyopathy (HCM), which is the most common inherited cardiovascular disease [6]. Molecular mechanisms were extracted from abstracts and open access full articles by multiple machine-reading systems. Shared molecular mechanisms of HCM and its clinical presentations were represented as networks, and the most important elements in the networks were found based on node centrality measures.

The paper with the title “Impact of Water Temperature on Heart Rate Variability during Bathing” aims to explore the impact of water temperature (WT) on Heart Rate Variability (HRV) by collecting five electrocardiogram (ECG) recordings of each of 10 subjects at different preset bathtub WT conditions during bathing [7]. Based on statistical analysis, it has been shown that the WT has an important impact on HRV during bathing. 

The accuracy of several methods has been compared and assessed in the paper with the title “Discussion of Cuffless blood pressure prediction using plethysmograph based on a longitudinal experiment: Is individual model necessary?” [8]. Estimating blood pressure using the Plethysmograph (PPG) signal is convenient and makes continuous measurement feasible, but some doubt exists on its accuracy and robustness. By comparing the regression models, this paper shows that an individual Gaussian Process model attains the best results.
